# Comparative Effectiveness of Wearable Devices and Built-In Step Counters in Reducing Metabolic Syndrome Risk in South Korea: Population-Based Cohort Study

**DOI:** 10.2196/64527

**Published:** 2025-02-25

**Authors:** Kyung-In Joung, Sook Hee An, Joon Seok Bang, Kwang Joon Kim

**Affiliations:** ^1^School of AI Healthcare, College of Integrated Health Science, CHA University, Pocheon-si, Republic of Korea; 2 College of Pharmacy, Wonkwang University, Iksan, Republic of Korea; 3 College of Pharmacy, Sookmyung Women’s University, Seoul, Republic of Korea; 4 College of Pharmacy, Chonnam National University, BLDG1, Room No. 407, 77 Yongbong-ro, Gwangju, 61186, Republic of Korea, 82 01021120321, 82 625302949

**Keywords:** wearable devices, built-in step counters, mobile health, public health intervention, physical activity, health behavior, metabolic syndrome, population-based, cohort study, South Korea, mobile health technologies, effectiveness, activity tracker, mobile app, retrospective, logistic regression, mHealth, digital health, mobile phone

## Abstract

**Background:**

Mobile health technologies show promise in addressing metabolic syndrome, but their comparative effectiveness in large-scale public health interventions remains unclear.

**Objective:**

This study aims to compare the effectiveness of wearable devices (wearable activity trackers) and mobile app–based activity trackers (built-in step counters) in promoting walking practice, improving health behaviors, and reducing metabolic syndrome risk within a national mobile health care program operated by the Korea Health Promotion Institute.

**Methods:**

This retrospective cohort study analyzed data from 46,579 participants in South Korea’s national mobile health care program (2020‐2022). Participants used wearable devices for 12 weeks, after which some switched to built-in step counters. The study collected data on demographics, health behaviors, and metabolic syndrome risk factors at baseline, 12 weeks, and 24 weeks. Outcomes included changes in walking practice, health behaviors, and metabolic syndrome risk factors. Metabolic syndrome risk was assessed based on 5 factors: blood pressure, fasting glucose, waist circumference, triglycerides, and high-density lipoprotein cholesterol. Health behaviors included low-sodium diet preference, nutrition label reading, regular breakfast consumption, aerobic physical activity, and regular walking. To address potential selection bias, propensity score matching was performed, balancing the 2 groups on baseline characteristics including age, gender, education level, occupation, insurance type, smoking status, and alcohol consumption.

**Results:**

Both wearable activity tracker and built-in step counter groups exhibited significant improvements across all evaluated outcomes. The improvement rates for regular walking practice, health behavior changes, and metabolic syndrome risk reduction were high in both groups, with percentages ranging from 45.2% to 60.8%. After propensity score matching, both device types showed substantial improvements across all indicators. The built-in step counter group demonstrated greater reductions in metabolic syndrome risk compared to the wearable device group (odds ratio [OR] 1.20, 95% CI 1.05‐1.36). No significant differences were found in overall health behavior improvements (OR 0.95, 95% CI 0.83‐1.09) or walking practice (OR 0.84, 95% CI 0.70‐1.01) between the 2 groups. Age-specific subgroup analyses revealed that the association between built-in step counters and metabolic syndrome risk reduction was more pronounced in young adults aged 19‐39 years (OR 1.35, 95% CI 1.09‐1.68). Among Android use subgroups, built-in step counters were associated with a higher reduction in health risk factors (OR 1.20, 95% CI 1.03‐1.39).

**Conclusions:**

Both wearable devices and built-in step counters effectively reduced metabolic syndrome risk in a large-scale public health intervention, with built-in step counters showing a slight advantage. The findings suggest that personalized device recommendations based on individual characteristics, such as age and specific health risk factors, may enhance the effectiveness of mobile health interventions. Future research should explore the mechanisms behind these differences and their long-term impacts on health outcomes.

## Introduction

Metabolic syndrome, a cluster of interconnected factors, significantly increases the risk of cardiovascular disease, stroke, and type 2 diabetes, presenting a major challenge to global public health systems [1-3]. Its rising prevalence imposes substantial economic burdens on health care systems and society [4]. In response, digital health technologies, particularly mobile apps and wearable devices, have emerged as promising tools for health promotion and disease prevention. These technologies offer advantages such as real-time data collection, personalized feedback, and continuous monitoring, potentially enhancing user engagement and promoting sustainable behavior change to reduce metabolic syndrome risks [5,6].

Recent studies have demonstrated the potential of mobile health apps in supporting behavior change for diabetes prevention and control. A prospective cohort study in Singapore revealed that higher engagement with a mobile health app led to greater weight loss and HbA_1c_ reduction among adults with overweight or obesity and who had type 2 diabetes or prediabetes [7]. Similarly, a qualitative research highlighted the importance of integrating app use into routine care and developing guidelines for health care professionals to maximize the benefits of these technologies [8]. These findings underscore the growing importance of digital interventions in chronic disease management.

However, the efficacy of these digital interventions in public health settings remains a subject of debate. While some studies have reported significant benefits in health promotion and metabolic syndrome management [9,10], others have found limited or no effects [11,12]. This discrepancy underscores the need for further research to elucidate the factors that influence the effectiveness of digital health interventions in real-world settings.

Wearable devices are hypothesized to be more effective in promoting health behaviors due to their ability to provide continuous monitoring and immediate feedback, potentially enhancing user engagement and adherence to exercise regimens than mobile apps alone [10,13,14]. However, evidence supporting this hypothesis in large-scale public health interventions remains limited and inconsistent. Some studies suggest that wearables enhance physical activity and health monitoring more effectively than mobile apps due to their continuous tracking and user engagement features [15]. Conversely, other research indicates minimal differences in health outcomes between users of wearables and mobile apps, suggesting that the technology’s effectiveness may heavily rely on individual user engagement and integration into comprehensive health management strategies [16,17].

In South Korea, a nationwide mobile health care program operated by the Korea Health Promotion Institute (KHPI) has been operational since 2018, providing an unprecedented opportunity to evaluate the effectiveness of digital health interventions at a population level. This program, implemented through over 220 health centers, offers free wearable activity trackers to eligible participants to mitigate metabolic syndrome risks. Interestingly, some participants have opted to switch to using mobile phone built-in step counters due to personal preference or device loss, creating a natural experiment to compare the efficacy of these 2 approaches.

This study aims to comprehensively evaluate the comparative effectiveness of wearable devices (wearable activity trackers) versus mobile app–based activity trackers (built-in step counters) within this public health service framework. Specifically, we seek to examine their differential impacts on three key outcomes: (1) the promotion of regular walking practices, (2) improvements in overall health behaviors, and (3) the reduction of metabolic syndrome risk factors. By leveraging a large-scale, real-world dataset, we aim to provide nuanced insights into how these technologies influence various aspects of health behavior and outcomes.

## Methods

### Data Source

The data source is the KHPI’s health center mobile health care program data (2020‐2022). This nationwide program uses Information and Communication Technology to improve preventive health care and self-health capabilities of local residents aged 19 years and older with at least one metabolic syndrome risk factor. The program targets individuals who are generally healthy or without diagnosed chronic diseases. Participants undergo comprehensive health assessments at baseline, 3 months, and 6 months, supported by continuous mobile health care interventions throughout the 6-month program duration.

On enrollment, participants receive a mobile app and an activity tracker (wristband or smartwatch) that monitors biometric data, syncing with the app. The app features automated exercise and dietary logging, delivers personalized health information, and sends exercise and food diaries to participants. From week 12, participants could opt to use their smartphone’s built-in step counter instead of the provided tracker, integrating with fitness tracker apps.

### Study Design and Participants

**Figure 1.**
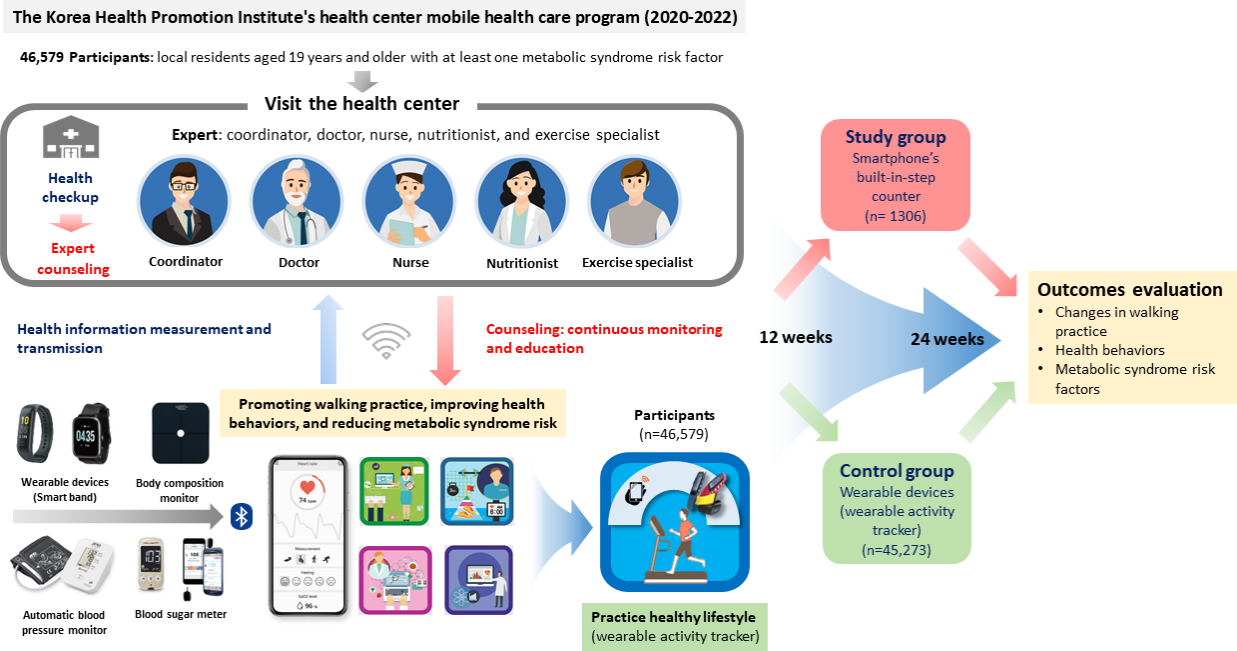
Health center mobile health care program and study design.

Participants receive medical check-ups, lifestyle assessments, and expert counseling at the beginning, week 12, and week 24. Exclusion criteria included: (1) nonparticipation for over 2 weeks, (2) onset of chronic diseases during the study, (3) incomplete final health questionnaire or pedometer loss by week 27, and (4) moving out of the service area. The Ministry of Health and Welfare oversees the program, with management by the KHPI (Figure 1) [18].

This retrospective cohort study examined changes in walking routines, health behavior patterns, and metabolic syndrome risk factors among participants who either continued using a wearable activity tracker or switched to a smartphone’s built-in step counter after 12 weeks of the program.

The study group comprised individuals with at least 1 metabolic syndrome risk factor who switched from a wearable activity tracker to their smartphone’s built-in step counter after 12 weeks. The control group consisted of individuals with at least 1 metabolic syndrome risk factor who continued using a wearable activity tracker throughout the 24-week intervention period.

### Outcome Measures

The practice of “regular walking” was defined based on a survey as engaging in walking for at least 10 minutes consecutively for 5 or more days in the past week. If individuals who did not practice “regular walking” before participating in the program started practicing it afterward, it was considered an improvement in “regular walking.”

Health behaviors were assessed through a comprehensive survey that evaluated 5 key indicators: preference for low-salt diets, regular breakfast consumption, attentiveness to nutritional labels, engagement in regular walking, and participation in aerobic exercises. If any of these 5 health behaviors were not practiced before the program but were adopted afterward, it was considered an improvement in health behaviors.

Metabolic syndrome risk factors were composed of the following five criteria, adopting the diagnostic criteria used domestically based on the National Cholesterol Education Program Adult Treatment Panel III guidelines: (1) elevated blood pressure (systolic blood pressure equal to or above 130 mm Hg or diastolic blood pressure equal to or above 85 mm Hg); (2) elevated fasting blood glucose equal to or above 100 mg/dL; (3) abdominal obesity: male waist circumference equal to or above 90 cm, female waist circumference equal to or above 85 cm; (4) elevated triglycerides equal to or above 150 mg/dL; and (5) decreased high-density lipoprotein cholesterol: male less than 40 mg/dL, female less than 50 mg/dL. If 1 or more of these 5 metabolic syndrome risk factors decreased after participation in the program compared to before, it was considered a reduction in metabolic syndrome risk [19].

### Potential Confounding Variables

The following potential confounding variables were included as covariates: gender, age group (in 10 y increments), BMI, education level (4 levels), occupation, family type, insurance type (as a proxy for economic status), smoking, alcohol consumption, and mobile operating system.

### Statistical Analysis

Baseline demographic characteristics were compared between the built-in step counter users and wearable activity tracker users using chi-square tests. To enhance comparability between the study group and the control group, we performed propensity score matching as our primary analysis method [20]. Propensity scores were estimated using multivariable logistic regression for all variables mentioned earlier. Matching was performed using a 1:4 nearest neighbor method with the estimated propensity score, and the caliper was strictly set at 0.05. The standardized mean difference was used to compare baseline characteristics of the study group and control group, with imbalance defined as an absolute value greater than 0.1.

Using the propensity score-matched data, we assessed the associations between device type and (1) regular walking practice, (2) health behavior improvement, and (3) metabolic syndrome risk reduction. Odds ratios (OR) with 95% CIs were calculated to quantify these associations.

As a secondary analysis, we conducted multivariable logistic regression with backward elimination, adjusting for potential confounding variables, to further validate our findings. This analysis was performed on the full dataset before propensity score matching.

Subgroup analyses were performed based on age groups (10-30 years: young adults; 40-50 years: middle-aged adults; and 60 years and older: older people) and mobile operating systems (Android and iOS) using both the propensity score-matched data and the full dataset with multivariable logistic regression.

### Ethical Considerations

This study was approved by the Bioethics Committee of the CHA University Institutional Review Board (approval E1705/001-003). This study used secondary data from the Public Health Center Mobile Health Care Project, a public health initiative in South Korea. The institutional review board deemed this study exempt from the requirement for additional informed consent, as it involved the secondary analysis of deidentified data from an existing public health program. All data were anonymized and deidentified by the public health centers before being provided for research purposes, with all personal identifiers removed to ensure participant privacy and confidentiality. The data management and analysis procedures strictly adhered to the Personal Information Protection Act of South Korea and institutional data security protocols. As this was a secondary analysis of data from a public health program, participants were not provided additional compensation for this specific research study. Their original participation in the Mobile Health Care Project was voluntary and part of a public health service initiative, without monetary compensation.

## Results

Compared to continuous wearable users (n=45,273, 97.2%), the proportion of users who switched to built-in step counters was small (n=1306, 2.8%). The built-in step counter group had a higher proportion of younger adults (19‐39 y), iOS users, and those with higher education levels. This group also included more managers, professionals, and office workers. In contrast, housewives were more likely to continue using wearable devices. Men showed a slightly higher proportion of built-in step counter use compared to women (n=486, 3.2% vs n=820, 2.6%). Among those with a Bachelor’s degree or higher, built-in step counter use was slightly more common than wearable activity tracker use (n=1037, 79.4% vs n=33,659, 74.3%; Table 1).

**Table 1.** Basic characteristics of the participants by device type.

Variable and class		Wearable activity tracker (n=45,273, 97.2%)	Built-in step counter (n=1306, 2.8%)	Overall	*P* value
Sex, n (%)					<.001
	Male	14,800 (32.7)	486 (37.2)	15,286 (32.8)	
	Female	30,473 (67.3)	820 (62.8)	31,293 (67.2)	
Age group (years), n (%)					<.001
	10-19	79 (0.2)	3 (0.2)	82 (0.2)	
	20-29	3034 (6.7)	163 (12.5)	3197 (6.9)	
	30-39	9652 (21.3)	357 (27.3)	10,009 (21.5)	
	40-49	16,850 (37.2)	426 (32.6)	17,276 (37.1)	
	50-59	12,509 (27.6)	280 (21.4)	12,789 (27.5)	
	60-69	2937 (6.5)	70 (5.4)	3007 (6.5)	
	70-79	206 (0.5)	7 (0.5)	213 (0.5)	
	80-89	6 (0)	0 (0)	6 (0)	
mOS^a^, n (%)					<.001
	Android	40,208 (88.8)	976 (74.7)	41,184 (86.7)	
	iOS	5062 (11.2)	330 (25.3)	5392 (13.3)	
Family type, n (%)					.030
	Single person	3727 (8.2)	134 (10.3)	3861 (8.3)	
	2‐3 members	20,532 (45.4)	573 (43.9)	21,105 (45.3)	
	≥4 members	21,014 (46.4)	599 (45.9)	21,613 (46.4)	
Insurance type, n (%)					.363
	Health insurance	44,652 (98.6)	1287 (98.6)	45,939 (98.6)	
	Medical aid	359 (0.8)	8 (0.6)	367 (0.8)	
	Not enrolled	262 (0.6)	11 (0.8)	273 (0.6)	
Education, n (%)					<.001
	No education to middle school graduate	1037 (2.3)	19 (1.5)	1056 (2.3)	
	High school graduate	10,577 (23.4)	250 (19.1)	10,827 (23.2)	
	Bachelor’s degree	29,340 (64.8)	900 (68.9)	30,240 (64.9)	
	Master’s degree or higher	4319 (9.5)	137 (10.5)	4456 (9.6)	
Occupation, n (%)					<.001
	Manager, expert	9301 (20.5)	319 (24.4)	9620 (20.7)	
	Office worker, service worker	19,542 (43.2)	579 (44.3)	20,121 (43.2)	
	Sales people, agricultural, forestry, and fishery workers, technician, machine operator, elementary occupation, military personnel	5374 (11.9)	140 (10.7)	5514 (11.8)	
	Student	661 (1.5)	25 (1.9)	686 (1.5)	
	Homemaker	8944 (19.8)	193 (14.8)	9137 (19.6)	
	Unemployed	1451 (3.2)	50 (3.8)	1501 (3.2)	
Smoking, n (%)					.005
	No smoking	36,630 (80.9)	1031 (78.9)	37,661 (80.9)	
	Used to smoke but currently do not smoke	5037 (11.1)	146 (11.2)	5183 (11.1)	
	Smoke occasionally	815 (1.8)	37 (2.8)	852 (1.8)	
	Smoke every day	2791 (6.2)	92 (7)	2883 (6.2)	
Alcohol intake, n (%)					.61
	<0‐1 times/month	20,632 (45.6)	558 (42.7)	21,190 (45.5)	
	2‐4 times/month	16,768 (37)	486 (37.2)	17,254 (37)	
	2‐3 times/week	6304 (13.9)	211 (16.2)	6515 (14)	
	≥4 times/week	1569 (3.5)	51 (3.9)	1620 (3.5)	
General or intensive management needed^c^, n (%)					.98
	General	29,584 (65.4)	853 (65.3)	30,437 (65.3)	
	Intensive	15,689 (34.7)	453 (34.7)	16,142 (34.7)	
BMI^b^ (kg/m^2^), mean (SD)		25.4 (3.8)	25.7 (4)	25.4 (3.8)	.01
Waist circumference (cm), mean (SD)					
	Male	92.6 (8.9)	93.7 (9.8)	92.6 (9.0)	.02
	Female	85.2 (9.6)	84.5 (10.2)	85.1 (9.6)	.06
Blood pressure (mm Hg), mean (SD)					
	Systolic	126.3 (13.5)	125.4 (13.4)	126.3 (13.5)	.01
	Diastolic	80.4 (10.4)	79.8 (10.5)	80.4 (10.4)	.06
FBG^d^ (mg/dL), mean (SD)		98.0 (13.2)	97.8 (11.9)	98.0 (13.1)	.60
Triglyceride (mg/dL), mean (SD)		150.0 (84.9)	150.0 (80.9)	150.0 (84.8)	.99
HDL^e^ cholesterol (mg/dL), mean (SD)		55.7 (14.6)	54.9 (14.6)	55.7 (14.6)	.07

a mOS: mobile operating system.

b BMI: body mass index

c Individuals exhibiting 1-2 metabolic syndrome risk factors were allocated to a standard management group, whereas those presenting with 3-5 risk factors were assigned to an intensive management group.

d FBG: fasting blood glucose.

e HDL: high-density lipoprotein.

Overall, 54% (n=25,158) of participants showed a reduction in metabolic syndrome risk, with women demonstrating a higher rate than men (n=17,371, 55.5% vs n=7787, 50.9%). The reduced risk group had slightly higher proportions of young adults (10‐39 years), built-in step counter users, and iOS users. Participants with healthier baseline lifestyle indicators (nonsmoking, lower alcohol consumption, and lower BMI) were more prevalent in the reduced-risk group. The proportion of individuals showing a reduction in metabolic syndrome risk was slightly higher among those who practiced fewer health behaviors at baseline (Table 2).

**Table 2.** Characteristics of the participants by the reduction in metabolic syndrome risk.

Characteristic		No metabolic syndrome risk reduction (n=21,421, 46%), n (%)	Metabolic syndrome risk reduction (n=25,158, 54%), n (%)	Overall (n=46,579, 100%), n (%)	*P* value
Sex					<.001
	Male	7499 (35)	7787 (31)	15,286 (32.8)	
	Female	13,922 (46)	17,371 (69.1)	31,293 (67.2)	
Age (years)					<.001
	10‐29	1334 (6.2)	1945 (7.7)	3279 (7)	
	30-39	4612 (21.5)	5397 (21.5)	10,009 (21.5)	
	40-49	7979 (37.3)	9297 (37)	17,276 (37.1)	
	50-59	5912 (27.6)	6877 (27.3)	12,789 (27.5)	
	60-69	1470 (6.9)	1537 (6.1)	3007 (6.5)	
	70‐89	114 (0.5)	105 (0.4)	219 (0.5)	
Family type					.47
	Single person	1749 (8.2)	2112 (8.4)	3861 (8.3)	
	2‐3 members	9762 (45.6)	11,343 (45.1)	21,105 (45.3)	
	≥4 members	9910 (46.3)	11,703 (46.5)	21,613 (46.4)	
Education					.03
	No education to middle school graduate	508 (2.4)	548 (2.2)	1056 (2.3)	
	High school graduate	5062 (23.6)	5765 (22.9)	10,827 (23.2)	
	Bachelor’s degree	13,762 (64.3)	16,478 (54.5)	30,240 (64.9)	
	Master’s degree or higher	2089 (9.8)	2367 (9.4)	4456 (9.6)	
Insurance					.79
	Health insurance	21,125 (98.6)	24,814 (98.6)	45,939 (98.6)	
	Medical aid	174 (0.8)	193 (0.8)	367 (0.8)	
	Not enrolled	122 (0.6)	151 (0.6)	273 (0.6)	
Device type					.007
	Wearable	20,868 (97.4)	24,405 (97)	45,273 (97.2)	
	Switched to app	553 (2.6)	753 (3)	1306 (2.8)	
mOS^a^					<.001
	Android	19,097 (89.2)	22,087 (87.8)	41,184 (88.4)	
	iOS	2323 (10.9)	3069 (12.2)	5392 (11.6)	
Smoking					<.001
	No smoking	17,020 (79.5)	20,641 (82.1)	37,661 (80.9)	
	Used to smoke but currently do not smoke	2516 (11.8)	2667 (10.6)	5183 (11.1)	
	Smoke occasionally	426 (2)	426 (1.7)	852 (1.8)	
	Smoke every day	1459 (6.8)	1424 (5.7)	2883 (6.2)	
Alcohol intake					.001
	<0‐1 times/month	9597 (44.8)	11,593 (46.1)	21,190 (45.5)	
	2‐4 times/month	7970 (37.2)	9284 (36.9)	17,254 (37)	
	2‐3 times/week	3073 (14.4)	3442 (13.7)	6515 (14)	
	≥4 times/week	781 (3.7)	839 (3.3)	1620 (3.5)	
BMI					<.001
	<18.5	221 (1)	381 (1.5)	602 (1.3)	
	18.5‐23	4597 (21.5)	6657 (26.5)	11,254 (24.2)	
	23‐25	4346 (20.3)	5887 (23.4)	10,233 (22)	
	25‐30	9176 (42.8)	9849 (39.2)	19,025 (40.9)	
	30‐35	2551 (11.9)	1998 (7.9)	4549 (9.8)	
	≥35	530 (2.5)	385 (1.5)	915 (2)	
Number of metabolic syndrome risk factors at baseline					<.001
	1	9439 (44.1)	6784 (27)	16,223 (34.8)	
	2	6705 (31.3)	7509 (29.9)	14,214 (30.5)	
	3	3641 (17)	6176 (24.6)	9817 (21.1)	
	4	1420 (6.6)	3602 (14.3)	5022 (10.8)	
	5	216 (1)	1087 (4.3)	1303 (2.8)	
Hypertension risk					<.001
	Yes	10,157 (47.4)	14,846 (59)	25,003 (53.7)	
	No	11,264 (52.6)	10,312 (41)	21,576 (46.3)	
Diabetes mellitus risk					<.001
	Yes	7079 (33.1)	11,379 (45.2)	18,458 (39.6)	
	No	14,342 (67)	13,779 (54.8)	28,121 (60.4)	
High waist circumference					.16
	Yes	12,298 (57.4)	14,607 (58.1)	26,905 (57.8)	
	No	9123 (42.6)	10,551 (42)	19,674 (42.2)	
High TG^b^					<.001
	Yes	6703 (31.3)	11,806 (46.9)	18,509 (39.7)	
	No	14,718 (68.7)	13,352 (53.1)	28,070 (60.3)	
Low HDL^c^ cholesterol					
	Yes	4295 (20.1)	7535 (30)	11,830 (25.4)	
	No	17,126 (80)	17,623 (70.1)	34,749 (74.6)	
Number of health behaviors being practicing at baseline					<.001
	0	221 (1)	381 (1.5)	602 (1.3)	
	1	4597 (21.5)	6657 (26.5)	11,254 (24.2)	
	2	4346 (20.3)	5887 (23.4)	10,233 (22)	
	3	9176 (42.8)	9849 (39.2)	19,025 (40.9)	
	4	2551 (11.9)	1998 (7.9)	4549 (9.8)	
	5	530 (2.5)	385 (1.5)	915 (2)	

a mOS: mobile operation system.

b TG: triglyceride.

c HDL: high density lipoprotein.

After ensuring comparability between the wearable activity tracker and built-in step counter groups through propensity score matching, an analysis was conducted. Before matching, there were differences in various variables such as age, insurance type, and education level. However, after matching, the absolute values of the standardized mean differences were all below 0.1, indicating no significant differences between the 2 groups (Multimedia Appendix 1).

Table 3 presents the results of the propensity score-matched analysis. For regular walking practice, there was no significant difference between the 2 groups (OR 0.84, 95% CI 0.70‐1.01). Similarly, no significant differences were found in overall health behavior improvements (OR 0.95, 95% CI 0.83‐1.09).

Notably, the built-in step counter group demonstrated greater reductions in metabolic syndrome risk compared to the wearable device group (OR 1.20, 95% CI 1.05‐1.36). This significant association was particularly pronounced among young adults aged 10‐30 years (OR 1.35, 95% CI 1.09‐1.68). The effect was less pronounced and not statistically significant for the 40‐50 years age group (OR 1.15, 95% CI 0.97‐1.38) and the 60‐80 years age group (OR 1.08, 95% CI 0.61‐1.94).

When analyzed by the mobile operating system, Android users showed a significant association between built-in step counter use and metabolic syndrome risk reduction (OR 1.20, 95% CI 1.03‐1.39). For iOS users, the association was in the same direction but not statistically significant (OR 1.14, 95% CI 0.88‐1.50), possibly due to the smaller sample size in this subgroup (Table 3).

**Table 3.** Propensity score-matched analysis of device type association with walking practice, health behaviors, and metabolic syndrome risk reduction.

Study population and number of participants by device type					Propensity score matched model OR^a^ (95% CI)
Not practicing regular walking at baseline^b^					
	Wearable activity tracker (n=2468, 79.9%)				reference
	Built-in step counter (n=621, 20.1%)				0.84 (0.70‐1.01)
Not practicing any health behaviors at baseline^c^					
	Wearable activity tracker (n=4951, 80%)				reference
	Built-in step counter (n=1244, 20%)				0.95 (0.83‐1.09)
One or more metabolic syndrome risk factors at baseline^d^					
	Overall				
		Wearable activity tracker (n=5195, 80%)			reference
		Built-in step counter (n=1308, 20%)			1.20 (1.05‐1.36)
	10‐39 years				
		Wearable activity tracker (n=2035, 83%)			reference
		Built-in step counter (n=516, 17%)			1.35 (1.09‐1.68)
	40‐59 years				
		Wearable activity tracker (n=2808, 80%)			reference
		Built-in step counter (n=704, 20%)			1.15 (0.97‐1.38)
	60‐89 years				
		Wearable activity tracker (n=288, 79.9%)			reference
		Built-in step counter (n=75, 20.1%)			1.08 (0.61‐1.94)
	Android				
		Wearable activity tracker (n=3893, 80%)			reference
		Built-in step counter (n=975, 20%)			1.20 (1.03‐1.39)
	iOS				
		Wearable (n=1250, 79.3%)			reference
		Built-in step counter (n=326, 21.7%)			1.14 (0.88‐1.50)

a OR: odds ratio.

b Outcome: practicing walking.

c Outcome: improved in 1 or more health behaviors.

d Outcome: reduced in 1 or more metabolic syndrome risk factors.

Across all outcomes—regular walking practice, health behavior improvements, and metabolic syndrome risk reduction—both wearable activity tracker and built-in step counter groups showed high improvement rates, ranging from 45.2% to 60.8% (Table 4).

**Table 4.** Sensitivity analysis: multivariable analysis of device type association with walking practice, health behaviors, and metabolic syndrome risk reduction.

Study population and number of participants by device type				Improved, n (%)	Crude model, OR^a^ (95% CI)	Covariates model I^b^, OR (95% CI)	Covariates model II^c^, OR (95% CI)
Not practicing regular walking at baseline^d^							
	Wearable activity tracker (n=22,795, 97.3%)			11,705 (51.3)	reference	reference	reference
	Built-in step counter (n=624, 2.7%)			282 (45.2)	0.78 (0.67‐0.92)	0.82 (0.70‐0.96)	0.83 (0.66‐1.06)
Not practicing any health behaviors at baseline^e^							
	Wearable activity tracker (n=43,666, 96.5%)			26,569 (60.8)	reference	reference	reference
	Built-in step activity tracker (n=1253, 2.8%)			730 (58.3)	0.89 (0.80‐1.00)	0.92 (0.82‐1.03)	0.94 (0.84‐1.04)
One or more metabolic syndrome risk factors at baseline^f^							
	Wearable activity tracker (n=45,273, 97.2%)			24,405 (53.9)	reference	reference	reference
	Built-in step counter (n=1306, 2.8%)			753 (57.7)	1.16 (1.04‐1.30)	1.16 (1.04‐1.29)	1.15 (1.03‐1.30)

a OR: odds ratio.

b Covariates model Ⅰ: adjusted for age, sex.

c Covariates model Ⅱ: adjusted for age, sex, education, family type, insurance type, occupation, BMI, smoking, alcohol intake, mobile operation system type.

d Outcome: regular walking practice.

e Outcome: improved in 1 or more health behaviors.

f Outcome: reduced in 1 or more metabolic syndrome risk factors.

To further validate our findings, we conducted multivariable logistic regression analyses, presented in Table 4. These results were generally consistent with the propensity score-matched analysis. For regular walking practice, wearable activity trackers showed a slight advantage over built-in step counters in the crude model (OR 0.78, 95% CI 0.67‐0.92). However, this association weakened after adjusting for multiple covariates (fully adjusted OR 0.83, 95% CI 0.66‐1.06). No significant association was found between device type and overall health behavior improvement across all models (fully adjusted OR 0.94, 95% CI 0.84‐1.04). Interestingly, the use of built-in step counters was associated with greater reductions in metabolic syndrome risk compared to wearable activity trackers. This relationship remained consistent and statistically significant across all 3 models (crude OR 1.16, 95% CI 1.04‐1.30; fully adjusted OR 1.15, 95% CI 1.03‐1.30; Table 4).

Table 5 presents the results of subgroup analyses using multivariable logistic regression. These analyses revealed nuanced differences across age groups and mobile operating systems. Wearable activity trackers were significantly associated with better improvements in regular walking practice only in the 40-59 year old group (OR 0.80, 95% CI 0.64‐0.99). The device type did not significantly impact health behavior improvement across any age group. For metabolic syndrome risk, built-in step counters were significantly associated with greater risk reduction only among young adults (10-39 years; OR 1.21, 95% CI 1.01‐1.45), aligning with the findings from the propensity score-matched analysis.

In analyses by the mobile operating system, Android users showed a significant association between built-in step counter use and (1) less regular walking practice (OR 0.81, 95% CI 0.67‐0.97), and (2) metabolic syndrome risk reduction (OR 1.13, 95% CI 1.00‐1.29). iOS users demonstrated no significant differences between device types for any outcome, possibly due to limited statistical power.

**Table 5.** Sensitivity analysis: multivariable analysis in subgroup for the association of device type with regular walking practice, improvement in health behaviors, and metabolic syndrome risk reduction.

Study population, subgroup, and number of participants by device type			Covariates model II, OR^a^ (95% CI)
Not practicing walking at baseline^b^			
	10-39 years		
		Wearable activity tracker (n=6458, 96.1%)	reference
		Built-in step counter (n=261, 3.9%)	0.89 (0.69‐1.14)
	40-59 years		
		Wearable activity tracker (n=15,144, 97.8%)	reference
		Built-in step counter (n=337, 2.2%)	0.80 (0.64‐0.99)
	60-89 years		
		Wearable activity tracker (n=1193, 97.9%)	reference
		Built-in step counter 26 (2.1)	0.61 (0.27‐1.37)
	Android		
		Wearable activity tracker (n=20,287, 97.7%)	reference
		Built-in step counter (n=477, 2.3%)	0.81 (0.67‐0.97)
	iOS		
		Wearable activity tracker (n=2507, 94.5%)	reference
		Built-in step counter (n=147, 5.5%)	0.89 (0.63‐1.25)
Not practicing any health behaviors at baseline^c^			
	10-39 years		
		Wearable activity tracker (n=12,270, 96.1%)	reference
		Built-in step counter (n=505, 4%)	0.86 (0.72‐1.03)
	40-59 years		
		Wearable activity tracker (n=27,856, 97.7%)	reference
		Built-in step counter (n=668, 2.3%)	1.00 (0.85‐1.17)
	60-89 years		
		Wearable activity tracker (n=2788, 97.5%)	reference
		Built-in step counter (n=73, 2.5%)	0.72 (0.45‐1.17)
	Android		
		Wearable activity tracker (n=38,190, 97.6%)	reference
		Built-in step counter (n=932, 2.4%)	0.94 (0.83‐1.08)
	iOS		
		Wearable activity tracker (n=4811, 93.9%)	reference
		Built-in step counter (n=314, 6.1%)	0.85 (0.67‐1.07)
One or more Metabolic syndrome risk factors at baseline^d^			
	10-39 years		
		Wearable activity tracker (n=12,765, 96.1%)	reference
		Built-in step counter (n=523, 3.9%)	1.21 (1.01‐1.45)
	40-59 years		
		Wearable activity tracker (n=29,359, 97.7%)	reference
		Built-in step counter (n=706, 2.4%)	1.11 (0.96‐1.29)
	60-89 years		
		Wearable activity tracker (n=3149, 97.2%)	reference
		Built-in step counter (n=77, 2.8%)	1.17 (0.74‐1.86)
	Android		
		Wearable activity tracker (n=40,208, 97.6%)	reference
		Built-in step counter (n=976, 2.4%)	1.13 (1.00‐1.29)
	iOS		
		Wearable activity tracker (n=5062, 93.9%)	reference
		Built-in step counter (n=330, 6.1%)	1.18 (0.94‐1.49)

a OR: odds ratio.

b Outcome: regular walking practice.

c Outcome: improved in 1 or more health behaviors.

d Outcome: reduced in 1 or more metabolic syndrome risk factors.

## Discussion

### Principal Results

This study compared the effects of continuously using wearable devices versus switching to built-in step counters after 12 weeks on walking practice, health behaviors, and metabolic syndrome risk reduction. Both groups showed substantial improvements across all indicators, with 45%‐61% of participants demonstrating positive changes.

Using propensity score-matched analysis, we found that the group that switched to built-in step counters showed better results in metabolic syndrome risk reduction (OR 1.20, 95% CI 1.05‐1.36). This effect was particularly pronounced among young adults aged 10‐30 years (OR 1.35, 95% CI 1.09‐1.68). There were no significant differences between groups in walking practice (OR 0.84, 95% CI 0.70‐1.01) or overall health behaviors (OR 0.95, 95% CI 0.83‐1.09).

Age-specific subgroup analyses revealed that the effects of device type varied across age groups, with the association between switching to built-in step counters and metabolic syndrome risk reduction being more pronounced in young adults (10-30 years).

### Comparison With Previous Studies

Our research findings contribute to the evidence supporting the effectiveness of mobile technology in promoting physical activity and health behaviors. The substantial improvements observed across all indicators in both groups align with previous studies demonstrating the positive impact of smartphone apps and activity trackers on physical activity levels and health outcomes [17,21-24]. Recent research further supports this, showing that higher engagement with mobile health apps led to greater weight loss and HbA_1c_ reduction in adults with overweight or obesity and type 2 diabetes or prediabetes [7].

Our analysis showed no significant difference in walking practice between wearable device users and built-in step counter users (OR 0.84, 95% CI 0.70‐1.01). This result contrasts with some previous studies demonstrating greater benefits of wearable devices. A randomized controlled trial (RCT) found that among inactive overweight or obese postmenopausal women, a Fitbit-based intervention significantly increased moderate to vigorous physical activity compared to standard pedometers (Fitbit: +62 min/week vs pedometer: –3 min/week; *P*=.01) [25]. Similarly, another RCT reported significantly higher daily average step counts in a group using both app and wearable devices compared to an app-only group (8165 vs 6034 steps; *P*=.02) [26]. The differences between our results and those of previous studies may stem from variations in study design, target population, and intervention duration. For instance, while Cadmus-Bertram et al [25] focused on a specific population (postmenopausal women) and measured moderate to vigorous physical activity as the primary outcome, this study included a broader population and evaluated walking practice and overall health behaviors.

Regarding health behavior improvements, our propensity score-matched analysis found no significant differences between the 2 groups (OR 0.95, 95% CI 0.83‐1.09). This partially aligns with findings from a previous study, which reported no difference in energy intake changes between mobile app and wearable “Bite Counter” device groups [27]. However, the same study observed a significant increase in physical activity in the wearable device group (mean 2015.4, SD 684.6 metabolic equivalent (METs) min/week; *P*=.02), which contrasts with our findings. Recent research found that both synchronous (videoconference) and asynchronous (prerecorded videos) digital interventions demonstrated good acceptability among inactive adults, suggesting various digital tools can effectively promote physical activity [28]. The importance of integrating app use into routine care has been highlighted, which may explain the similar improvements in both groups in this study [8]. These findings underscore the complexity of digital health interventions and the need for tailored approaches.

In metabolic syndrome risk reduction, our propensity score-matched analysis revealed significant decreases in both groups, with a slight advantage in the group that switched to built-in step counters (OR 1.20, 95% CI 1.05‐1.36). This contrasts with some previous studies, where mobile phone–based health coaching showed more effectiveness in improving metabolic syndrome–related indicators [29]. However, our results align with other studies demonstrating the potential of mobile software and wearable devices in reducing metabolic syndrome risk [9].

A notable aspect of our findings is the high rate of metabolic syndrome risk reduction (over 50%) in both groups, possibly due to the well-designed intervention characteristics of the national-level public health program. These results are similar to a 12-week RCT where the intervention group receiving physical activity feedback through wearable devices showed significant improvements in key clinical indicators of metabolic syndrome [22]. This supports the potential effectiveness of well-designed mobile health interventions in reducing metabolic syndrome risk.

However, mixed results are also present in the literature. Some studies report that mobile interventions have no significant impact on physical activity or health outcomes [12,30]. For instance, a study on overweight Taiwanese adults found that activity promotion systems provided short-term benefits for physical activity but did not significantly affect metabolic abnormalities [31]. Similarly, an RCT of multidimensional physical activity feedback intervention for primary care patients at risk of chronic diseases showed no effect on physical activity or health outcomes [32].

These conflicting results highlight the complexity of using technology for health risk management and physical activity promotion. The effectiveness of such interventions may depend on various factors, including intervention design, user engagement, and individual health status [9]. Our findings, along with these recent studies, highlight the potential of digital health interventions in chronic disease management. They suggest that the effectiveness of such interventions may not solely depend on the specific technology used (eg, wearable devices vs built-in step counters), but also on how well these technologies are integrated into comprehensive health programs and how they engage users in multiple aspects of health management. Our findings align with recent research on digital health interventions for chronic disease management. The mobile health care program incorporated gamification elements, defined as “the use of game design elements in nongame contexts” [33]. This was evident in features like national step count rankings and health behavior scores, which were designed to engage users and encourage healthier behaviors. These gamified components, along with performance-based rewards, likely enhanced user engagement and motivation. Our results support the effectiveness of gamification strategies in health interventions, consistent with findings from a systematic review on type 2 diabetes self-management, which reported improvements in key health outcomes, such as glycated hemoglobin levels and BMI [34]. This underscores the importance of considering these elements when implementing mobile health interventions in public health settings and suggests that while mobile health technologies show promise, their impact may vary across different contexts and populations.

### Potential Mechanisms and Explanations

The differential effects observed between wearable devices and built-in step counters in this study can be explained by several potential mechanisms. The marginal advantage of wearable devices in promoting walking activity may stem from their continuous and real-time data collection capabilities. These devices provide immediate feedback, which has been shown to enhance user engagement and motivation [15]. This instant feedback loop can heighten awareness of physical activity levels and encourage users to be more active throughout the day.

The noninferiority or similar health impact of built-in step counters compared to wearable trackers suggests that simplicity and accessibility play crucial roles in promoting health behaviors. This aligns with findings emphasizing that simple pedometers could be more effective than complex wearable trackers or smartphone apps in public health interventions. A meta-regression analysis of 57 RCTs revealed that body-worn trackers or smartphone apps were less effective than pedometers in interventions lasting less than 4 months (–834 steps/day; 95% CI –1542 to –126]) [35]. This underscores the potential value of straightforward, easily accessible tools in promoting physical activity within public health initiatives.

In this study, younger individuals, those with higher education, and iOS users showed higher rates of switching to built-in step counters. This may reflect these groups’ higher understanding and adherence, potentially relating to the slightly better health risk factor reduction observed in the built-in step counter group. However, the possibility of selection or residual bias should be considered.

Multivariable logistic regression analysis confirmed an association between built-in step counter use and greater metabolic syndrome risk reduction, though no difference was found in health behaviors between the 2 groups, and the strength of the association was weak. Subgroup analyses revealed differences only in specific groups, limiting result generalizability and suggesting the need for personalized interventions.

### Limitations

This study provides valuable insights into the real-world effectiveness of wearable devices and built-in step counters within a national public health program. Using a large-scale nationwide dataset enhances statistical power and generalizability. The application of diverse statistical methodologies, including propensity analysis, strengthens the reliability of the results.

However, several limitations should be noted. As an observational study, it has inherent limitations in establishing causality. The small number of participants who switched to built-in step counters may limit statistical power in some analyses. Reliance on self-reported data and the inability to assess long-term effects are significant limitations. Additionally, the study design, where all participants initially used wearable devices for 12 weeks before some switched to built-in step counters, limits the ability to compare effects from the intervention’s start. This design feature means that the effects of built-in step counters cannot be completely isolated from the potential carryover effects of initial wearable device use. Finally, our analysis may be limited by unmeasured confounding factors not available in our dataset.

### Implications and Future Research Directions

Our findings provide important implications for the design and implementation of public mobile health care initiatives. While wearable devices may offer some advantages in promoting physical activity, built-in step counters can be equally effective, particularly in reducing metabolic syndrome risk among young adults.

These results support a more personalized approach to device recommendations in public health programs, aligning with the growing trend toward personalized digital health interventions [36,37]. Programs could offer options between wearable devices and built-in step counters based on individual preferences, age, and other relevant factors, potentially improving intervention adherence and outcomes.

The high rate of metabolic syndrome risk reduction observed (over 50% in both groups) suggests that well-designed technology-supported public health interventions can significantly impact population health, especially when integrated into comprehensive national health programs.

Future research should explore the mechanisms behind age-related differences in device effectiveness. Longitudinal studies with longer follow-up periods could provide insights into the long-term effects of various device types on health outcomes, addressing gaps identified in previous research [38].

### Conclusions

This study compared the effectiveness of wearable devices and built-in step counters in a large-scale national mobile health care program. Both device types were effective in reducing metabolic syndrome risk, with the group switching to built-in step counters showing slightly better results (OR 1.20, 95% CI 1.05‐1.36). Wearable devices showed a marginal advantage in promoting walking practice, but there was no significant difference in overall health behavior improvement.

These findings suggest the importance of providing device selection options based on individual characteristics and preferences in public mobile health care programs. The high rate of metabolic syndrome risk reduction observed in both groups (over 50%) demonstrates the potential of well-designed mobile health interventions.

## Supplementary material

10.2196/64527 Multimedia Appendix 1 Standard mean differences before and after propensity score matching.

## Abbreviations

KHPI: Korea Health Promotion Institute
OR: odds ratio
RCT: randomized controlled trial
