# Supplementary material for: Comparative Effectiveness of Wearable Devices and Built-In Step Counters in Reducing Metabolic Syndrome Risk in South Korea: Population-Based Cohort Study
Source: JMIR Mhealth Uhealth. 2025 Feb 25;13:e64527. doi: 10.2196/64527 (PMC11878715; doi:10.2196/64527)
Supplement: Multimedia Appendix 1 [file mhealth-v13-e64527-s001.docx]

| Variable | **Class** | **Std. Mean Diff. in overall cohort (before matching)** | **Std. Mean Diff. in matched cohort (after matching)** |
| --- | --- | --- | --- |
| age | 10s | 0.012 | 0.020 |
|  | 20s | 0.175 | -0.014 |
|  | 30s | 0.135 | -0.015 |
|  | 40s | -0.098 | -0.004 |
|  | 50s | -0.151 | 0.029 |
|  | 60s | -0.05 | 0.003 |
|  | 70s | 0.011 | -0.001 |
|  | 80s | -0.012 | 0.000 |
| Sex | Male | 0.094 | 0.030 |
|  | Female | -0.094 | -0.030 |
| Family type | Living alone | 0.067 | -0.007 |
|  | two-person family | -0.036 | 0.012 |
|  | three-person family | 0.001 | 0.011 |
|  | Four-person family | -0.001 | -0.015 |
|  | ≥ Five-person family | 0.031 | -0.002 |
| Insurance type | Health Insurance (Regional) | 0.211 | 0.001 |
|  | Health Insurance (Employer-based) | -0.213 | 0.002 |
|  | Medical Aid Type 1 | -0.021 | 0.010 |
|  | Medical Aid Type 2 | -0.012 | -0.035 |
|  | Not enrolled | 0.029 | -0.004 |
| Education | No formal education | -0.020 | 0.000 |
|  | Elementary school graduate | -0.042 | -0.024 |
|  | Middle school graduate | -0.053 | -0.002 |
|  | High school graduate | -0.107 | 0.025 |
|  | University graduate | 0.089 | -0.033 |
|  | Postgraduate education | 0.031 | 0.023 |
| Occupation | Manager | 0.030 | 0.016 |
|  | Professional and Related Workers | 0.082 | 0.001 |
|  | Clerical Worker | 0.039 | -0.000 |
|  | Service Worker | -0.019 | 0.009 |
|  | Sales Worker | 0.004 | 0.005 |
|  | Agriculture, Forestry, and Fishery Worker | 0.020 | -0.009 |
|  | Technician and Related Support Worker | -0.009 | -0.004 |
|  | Machinery and Equipment Operator and Assembler | -0.016 | 0.012 |
|  | Unskilled Laborer | -0.100 | -0.019 |
|  | Military Personnel | 0.002 | -0.006 |
|  | Student | 0.033 | 0.017 |
|  | Homemaker | -0.140 | -0.024 |
|  | Unemployed | 0.032 | 0.008 |
| Initial check-up BMI | < 18.5 | 0.020 | 0.003 |
|  | 18.5-23 | -0.076 | -0.003 |
|  | 23-25 | 0.011 | 0.005 |
|  | 25-30 | 0.049 | 0.001 |
|  | 30-35 | -0.029 | -0.003 |
|  | ≥35 | 0.050 | -0.005 |
| Smoking | Smoke every day | 0.034 | -0.013 |
|  | Smoke occasionally | 0.062 | 0.000 |
|  | Used to smoke but currently do not smoke | 0.002 | 0.013 |
|  | No smoking | -0.048 | -0.002 |
| Alcohol intake | Less than once a month | -0.033 | 0.015 |
|  | About once a month | -0.028 | -0.020 |
|  | 2-4 times a month | 0.024 | -0.012 |
|  | 2-3 times a week | 0.061 | 0.019 |
|  | 4 or more times a week | 0.023 | -0.006 |
|  | Do not drink | -0.038 | 0.001 |
| Reading nutrition labels | No | -0.039 | 0.004 |
|  | Yes | 0.039 | -0.004 |
| Low-sodium diet | No | 0.122 | 0.015 |
|  | Yes | -0.122 | -0.015 |
| Having breakfast regularly | No | 0.091 | 0.006 |
|  | Yes | -0.091 | 0.006 |
| Engaging in physical activity | No | -0.026 | -0.002 |
|  | Yes | 0.026 | 0.002 |
| Walking regularly | No | -0.052 | -0.007 |
|  | Yes | 0.052 | 0.007 |
| Hypertension risk | No | 0.046 | -0.008 |
|  | Yes | -0.046 | 0.008 |
| Diabetes Mellitus risk | No | -0.012 | -0.044 |
|  | Yes | 0.012 | 0.044 |
| Abdominal obesity risk | No | 0.009 | 0.014 |
|  | Yes | -0.009 | -0.014 |
| Hypertriglyceridemia risk | No | -0.021 | -0.001 |
|  | Yes | 0.021 | 0.001 |
| Low HDL-C risk | No | -0.033 | 0.007 |
|  | Yes | 0.033 | -0.007 |
| Health risk group | Routine management - Basic | 0.012 | -0.035 |
|  | Routine management – Blood pressure | -0.006 | 0.004 |
|  | Routine management – Basic – Diabetes Mellitus | 0.018 | 0.020 |
|  | Routine management – Basic - Complex | -0.057 | -0.003 |
|  | Routine management - Basic | 0.014 | -0.005 |
|  | Routine management – Blood pressure | -0.037 | -0.025 |
|  | Routine management - Diabetes Mellitus | 0.036 | 0.020 |
|  | Routine management - Complex | 0.001 | 0.028 |
| OS | Android | -0.324 | 0.011 |
|  | iOS | 0.324 | -0.011 |
